# Safflower *CtFLS1*-Induced Drought Tolerance by Stimulating the Accumulation of Flavonols and Anthocyanins in *Arabidopsis thaliana*

**DOI:** 10.3390/ijms25105546

**Published:** 2024-05-19

**Authors:** Xintong Ma, Yuying Hou, Abdul Wakeel Umar, Yuhan Wang, Lili Yu, Naveed Ahmad, Na Yao, Min Zhang, Xiuming Liu

**Affiliations:** 1 Engineering Research Center of the Chinese Ministry of Education for Bioreactor and Pharmaceutical Development, College of Life Sciences, Jilin Agricultural University, Changchun 130118, China; mxt7142020@163.com (X.M.); houyy2017@163.com (Y.H.); 15944495496@163.com (Y.W.); 15640309810@163.com (L.Y.);; 2 BNU-HKUST Laboratory of Green Innovation, Advanced Institute of Natural Sciences, Beijing Normal University at Zhuhai (BNUZ), Zhuhai 519087, China; abwzju@zju.edu.cn; 3 Joint Center for Single Cell Biology, Shanghai Collaborative Innovation Center of Agri-Seeds, School of Agriculture and Biology, Shanghai Jiao Tong University, Shanghai 200240, China; naveedjlau@gmail.com; 4 Ginseng and Antler Products Testing Center of the Ministry of Agriculture PRC, Jilin Agricultural University, Changchun 130118, China

**Keywords:** anthocyanin, arabidopsis, CtFLS1, drought, flavonol, safflower

## Abstract

Flavonol synthase gene (*FLS*) is a member of the 2-oxoglutarate-dependent dioxygenase (*2-ODD*) superfamily and plays an important role in plant flavonoids biosynthetic pathways. Safflower (*Carthamus tinctorius* L.), a key source of traditional Chinese medicine, is widely cultivated in China. Although the flavonoid biosynthetic pathway has been studied in several model species, it still remains to be explored in safflower. In this study, we aimed to elucidate the role of *CtFLS1* gene in flavonoid biosynthesis and drought stress responses. The bioinformatics analysis on the *CtFLS1* gene showed that it contains two FLS-specific motifs (PxxxIRxxxEQP and SxxTxLVP), suggesting its independent evolution. Further, the expression level of *CtFLS1* in safflower showed a positive correlation with the accumulation level of total flavonoid content in four different flowering stages. In addition, *CtFLS1*-overexpression (OE) *Arabidopsis* plants significantly induced the expression levels of key genes involved in flavonol pathway. On the contrary, the expression of anthocyanin pathway-related genes and MYB transcription factors showed down-regulation. Furthermore, *CtFLS1*-OE plants promoted seed germination, as well as resistance to osmotic pressure and drought, and reduced sensitivity to ABA compared to mutant and wild-type plants. Moreover, CtFLS1 and CtANS1 were both subcellularly located at the cell membrane and nucleus; the yeast two-hybrid and bimolecular fluorescence complementation (BiFC) assay showed that they interacted with each other at the cell membrane. Altogether, these findings suggest the positive role of CtFLS1 in alleviating drought stress by stimulating flavonols and anthocyanin accumulation in safflower.

## 1. Introduction

Safflower plays a vital function in Chinese traditional medicine, nutrition, and dyes. The dried flowers of safflower have mainly been used to treat coronary heart disease, hypertension, cerebrovascular, and gynecological diseases and improve cerebral blood flow [1,2,3]. Flavonoids, especially the water-soluble constituents, play a pivotal role in producing these beneficial therapeutics. Among them, hydroxysafflor yellow A (HSYA), carthamin, and flavonols are predominant medicinally active components [4,5]. Meanwhile, flavonoids also play an important role in plant biotic and abiotic stresses, such as protection against light and temperature, as well as defense against insect infestation and pathogens [6,7,8]. In plants, flavonoids contribute to various pharmacological applications [9,10,11]. Recently, more than 10,000 flavonoid compounds were discovered and divided into nine subgroups: flavonols, anthocyanidins, flavanones, dihydro flavonols (DHFs), flavan-3,4-diols, flavones, flavan-3-ols, proanthocyanidins (PAs), and isoflavonoids [12,13].

**Figure 1:**
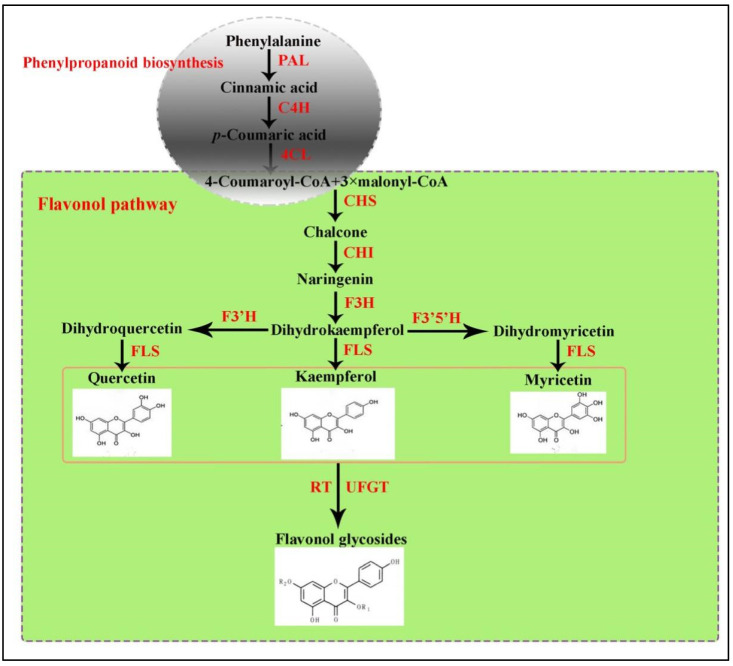
The core metabolic pathway of flavonol.

Flavonoids synthesize via the phenylpropanoid pathway, transforming phenylalanine into 4-coumaroyl-CoA, which subsequently enters the flavonoid biosynthesis pathway (Figure 1) [14]. Prior studies on flavonoids have shed light on their biosynthetic pathways and their significance in plant growth and development alongside health properties [15]. The chalcone scaffolds produced from the core flavonoid pathway undergo various configurations to produce several classes of compounds depending on the plant species and enzymes, such as hydroxylase, isomerases, reductases, and several Fe^2+^/2-oxoglutarate-dependent dioxygenases [12,16,17,18]. Anthocyanins typically exhibit a spectrum of colors (ranging from blue to purple to red). In contrast, flavonols, exemplified by kaempferol and quercetin, manifest a yellow hue. Apart from this, these predominant subgroup among flavonoids play pivotal roles in regulating growth, developmental, and protection/defense purposes [19,20,21].

During anthocyanins and flavonols biosynthesis, the dihydroflavonol compounds are synthesized by two different enzymes, namely dihydroflavonol 4-reductase (DFR) and/or FLS, respectively. Flavonol synthase (FLS, EC number 1.14.11.23) is a non-heme iron-containing soluble protein that belongs to the 2-oxoglutarate-dependent dioxygenase (2-ODD) superfamily [22,23]. It possesses two highly conserved domains, namely the DIOX_N domain (pfam14226) and the 2OG-FeII_Oxy domain (pfam03171) [24]. The first FLS cDNA was cloned from a *Petunia hybrida* and then extended to various plant species, including *Malus pumila* [25], *Paramecium caudatum* [26], *Litchi chinensis* [27], rice [28], *Brassica napus* [29], and *Arabidopsis thaliana* [30]. However, the identification and functional characterization *FLS* gene, (GenBank KP300884.1) have not been carried out yet.

In the present study, we aimed to investigate the functional characterization of *FLS1* gene in safflower. To this end, we cloned the full-length cDNA sequences of the *CtFLS1* gene followed by spatiotemporal expression analysis in different flowering stages. Additionally, the overexpression of *CtFLS1* in Arabidopsis was carried out to examine its regulatory mechanism under normal and stress conditions. We also conducted subcellular localization of CtFLS1, as well as yeast two-hybrid (Y2H) and bimolecular fluorescence complementation (BiFC) assays. Together, our findings suggest that CtFLS1 gene plays a key role in regulating flavonoid biosynthesis and drought tolerance in transgenic Arabidopsis. This study establishes a fundamental framework for elucidating and manipulating, CtFLS1 for future molecular breeding programs of safflower for enhanced flavonoid content and the amelioration of stress tolerance in adverse environmental conditions.

## 2. Results

### 2.1. Bioinformatics Analysis and Spatio-Temporal Expression of CtFLS1

**Figure 2:**
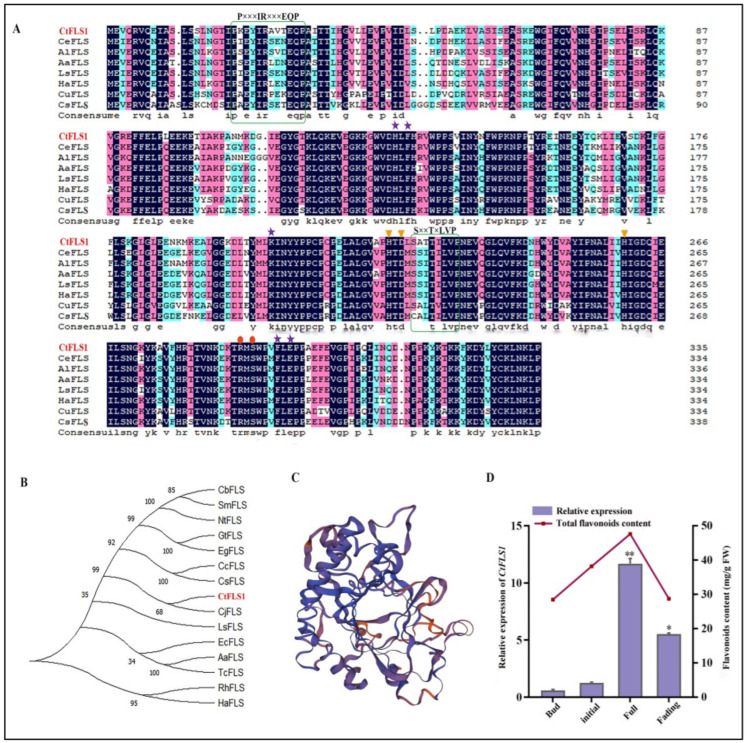
Bioinformatics analysis of CtFLS1 and expression analysis in four different flowering stages. (**A**) Multiple sequence alignment of CtFLS1 with that of other FLS proteins. The two green colored boxes highlight the FLS-specific motifs, PxxxIRxxxEQP and SxxTxLVP. Orange triangular markers indicate sites of amino acid residues critical for binding ferrous ions (H222, D224, and H260). Red circular markers represent the 2-oxoglutarate binding residues (R288 and S290). Purple asterisk shows the substrate binding residues (H133, F135, K205, F294, and E296). (**B**) CtFLS1 protein evolutionary tree constructed using the NJ method by MEGA-X software with 1000 bootstrap replicates. (**C**) Tertiary structure of CtFLS1 protein. (**D**) Expression analysis of the *CtFLS1* and the measurement of total flavonoid content at four flowering stages (bud, initial, full, and fading). Error bars indicate SE (*n* = 3). All quantitative data are expressed as mean values (x) ± variance (SD). * *p* < 0.05, ** *p* < 0.01.

The full length *CtFLS1* (GenBank: KP300884.1) sequence was cloned by RT-PCR, which consisted of a 1011 bp open reading frame encoding 336 amino acid residues. The isoelectric point CtFLS1 protein was 6.12, and the molecular weight was 38.21 kDa. To elucidate the function of CtFLS1 in safflower flavonoid biosynthesis, a homology search was conducted against FLS sequences from other plant species using BLAST. Comparison with known FLS sequences demonstrated significant identity, suggesting conservation of function across species (Figure 2A). Phylogenetic analysis further confirmed the evolutionary relationship between CtFLS1 and other FLS proteins, with CtFLS1 showing the highest similarity to *Cirsium japonicum* L. FLS (CjFLS) (Figure 2B). Additionally, the tertiary structure of CtFLS1 was predicted, providing insights into its functional characteristics (Figure 2C). Furthermore, the spatiotemporal expression analysis of CtFLS1 during different flowering stages of safflower, coupled with quantification of total flavonoid content, revealed a potential correlation between CtFLS1 transcript abundance and the accumulation trend of total flavonoids (Figure 2D). These findings contribute to our understanding of the molecular mechanisms underlying flavonoid biosynthesis in safflower and highlight the role of CtFLS1 as a key regulator in this pathway.

### 2.2. Overexpression of the CtFLS1 Directly Induce Flavonoid Biosynthesis Pathway in Transgenic Arabidopsis

To explore the likely role of *CtFLS1* gene in vivo, the plant overexpression vector (pBasta) driven by the CaMV 35S promoter was constructed with the *CtFLS1* CDS sequence and was transferred into wild-type (WT) col-0 Arabidopsis plants. Until T3 generation, the transgenic plants were collected and subject to expression analysis of *CtFLS1* gene. As shown in Figure S1, the relative expression level of *CtFLS1* was increased diversely in *CtFLS1*-overexpressing transgenic plants compared to WT, suggesting two highest expression peaks, including ~336.62-fold and ~192.67-fold observed in OE-1 and OE-7. Thus, these two T3 transgenic Arabidopsis lines were selected for further analysis. Furthermore, the expression analysis of flavonol pathway genes in transgenic plants and WT plants was carried out. The results showed that the overexpression of *CtFLS1* significantly enhanced the expression level of *AtFLS1* in OE lines when compared to WT plants. On the contrary, the expression levels of *AtDFR* and *AtANS* genes, which are implicated in the anthocyanin pathway, showed a significant decrease in the OE lines compared to the WT plants. Similarly, the expression levels of transcription factors, including *AtMYB11*, *AtMYB12*, *AtMYB111*, and *AtMYB113*, were found to be significantly lower in the OE lines as compared to WT.

**Figure 3:**
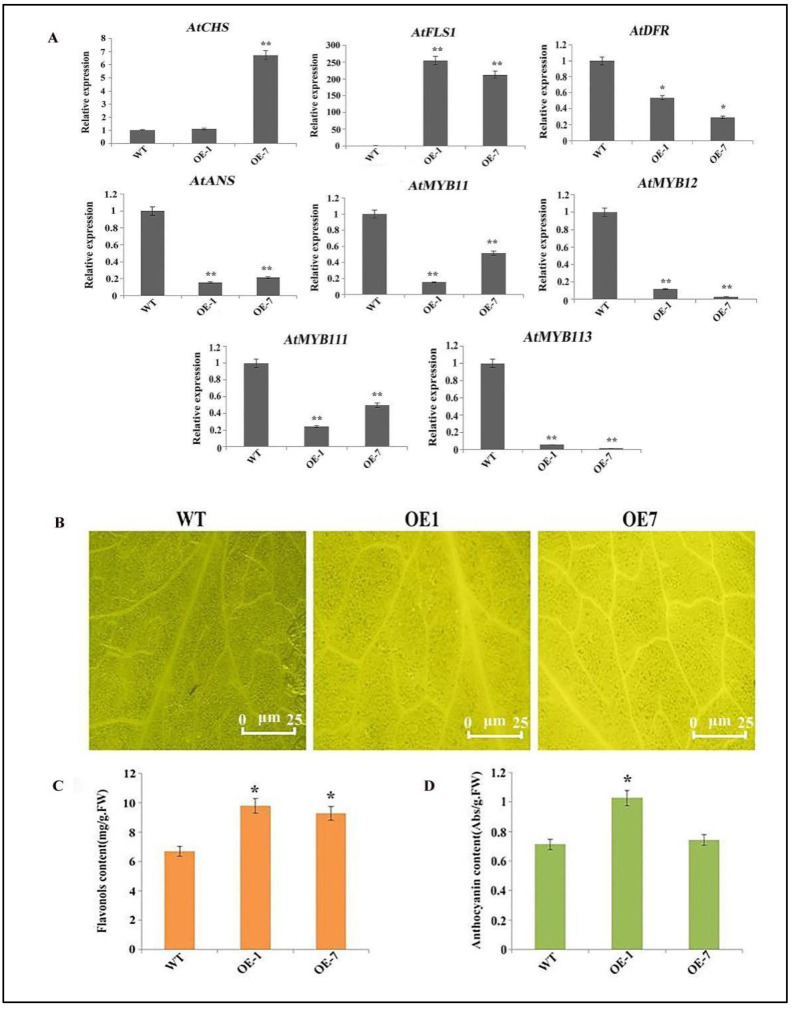
The overexpression of *CtFLS1* induced flavonoid biosynthesis pathway in Arabidopsis. (**A**) The expression pattern of the flavonoid biosynthesis pathway genes in the transgenic *Arabidopsis* lines. (**B**) Leaves of wild-type and *CtFLS1* transgenic Arabidopsis lines were used to detect flavonol content using DPBA staining. Bars = 25 μm. (**C**) Total flavonoids content in wild-type and transgenic Arabidopsis leaves. (**D**) Total anthocyanin content in wild-type and transgenic *Arabidopsis* leaves. FW, fresh weight. Significance analysis *t* test: * *p* < 0.05, ** *p* < 0.01.

To further explore the role of *CtFLS1* overexpression on the accumulation of flavonoids in transgenic Arabidopsis lines, we first detected total flavonoids and anthocyanin content in WT and transgenic Arabidopsis leaves. Total flavonoids content in WT and *CtFLS1*-overexpressing Arabidopsis leaves was significantly different (Figure 3C), suggesting that the leaves of OE-1 and OE-7 exhibited significantly higher total flavonoids content (9.78 mg/g and 9.26 mg/g FW) than WT leaves (6.69 mg/g FW). The total anthocyanin content in OE-1 leaves (1.026 Abs/g FW) was higher than in WT leaves (0.713 Abs/g FW), while the total anthocyanin content in OE-7 leaves (0.743 Abs/g FW) and WT leaves (0.713 Abs/g FW) were not significantly different (Figure 3D). Next, flavonol accumulation in the WT and transgenic Arabidopsis leaves were investigated using DPBA staining. Leaves displayed yellow fluorescence because of flavonol accumulation, as shown in Figure 3B, suggesting that flavonol accumulation levels in OE-1 and OE-7 leaves were higher than in WT leaves. Therefore, these results reveal that *CtFLS1*-overexpression could improve the accumulation of the flavonoid content and that *CtFLS1* is a key gene regulating flavonoid biosynthetic pathway in transgenic Arabidopsis lines.

### 2.3. CtFLS1 Enhanced Osmotic Stress Tolerance in Transgenic Arabidopsis

**Figure 4:**
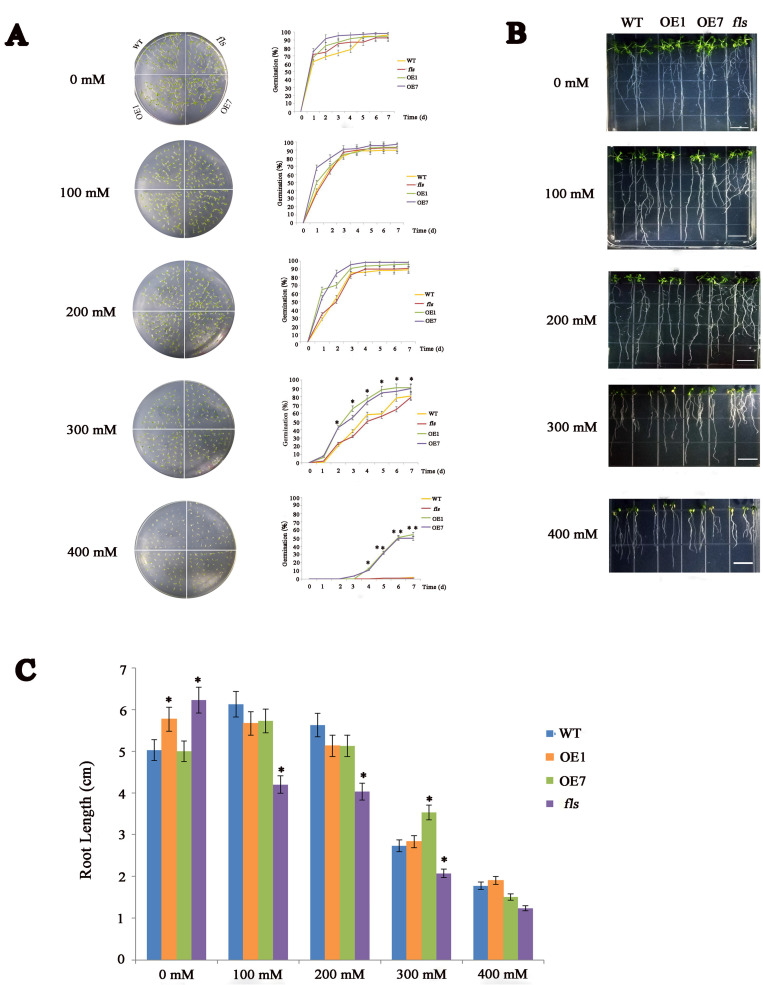
Phenotypic analysis of *CtFLS1* transgenic Arabidopsis under mannitol treatment. (**A**) Seed germinations of WT and *CtFLS1*-overexpressing lines under different concentrations of mannitol treatments. (**B**) Root lengths phenotype of wild type and *CtFLS1* transgenic Arabidopsis seedlings grown on 1/2 MS medium with or without mannitol for 10 days. (**C**) Statistics of root length. Data are means ± standard deviations from three replicates. * *p* < 0.05, ** *p* < 0.01.

To investigate the function of *CtFLS1* in response to osmotic stress, the seeds of WT, *fls* mutant and overexpression (OE1 and OE7) lines were sown on 1/2 MS medium and 1/2 MS medium containing 100 mM, 200 mM, 300 mM, and 400 mM mannitol. The seed germination rate was monitored continuously for 7 days. It was found that there was no significant difference in the germination rates among these lines under normal conditions. However, in the presence of 300 mM mannitol, the germination rate of OE lines was over 80% on the fifth day compared to less than 60% of the WT and fls mutant lines. Meanwhile, in the presence of 400 mM mannitol, the germination rate of OE lines was nearly 50% on the seventh day, but the seeds of WT and fls mutant showed almost no germination at the same time (Figure 4A).

To further assess the sensitivity of *CtFLS1* OE plants to osmotic stress, root elongation inhibition was also analyzed under osmotic stress. The WT, *fls* mutant, and overexpression (OE1 and OE7) plant seedlings (5-day-old) were transferred to 1/2 MS medium and 1/2 MS medium containing 100 mM, 200 mM, 300 mM, and 400 mM mannitol. After 10 days, although the seedlings of both the transgenic and WT plants grew weakly and the rosette leaves turned yellow under 300 mM mannitol treatment conditions, the root lengths of transgenic plants were longer than those of WT plants (Figure 4B,C). Taken together, these results showed that *CtFLS1* transgenic lines demonstrated stronger capacity to resist osmotic stress.

### 2.4. Overexpression of CtFLS1 in A. thaliana Reduces the Sensitivity to ABA

**Figure 5:**
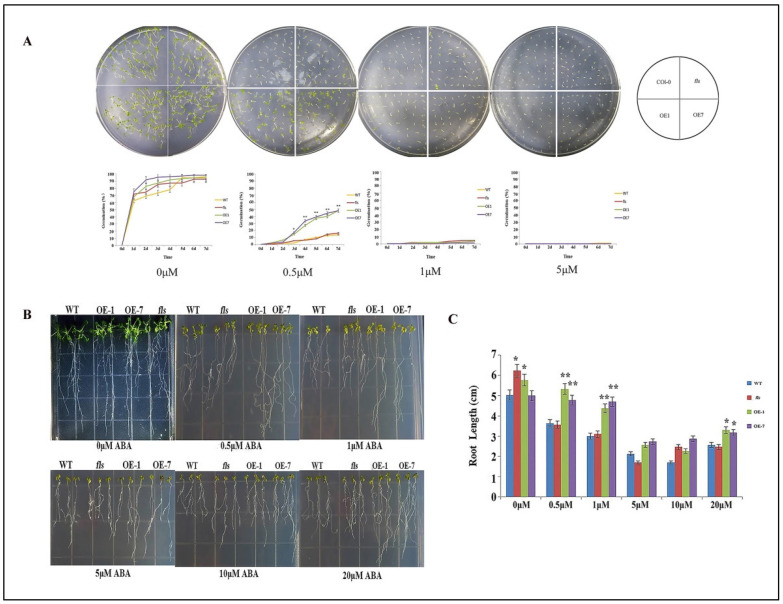
Sensitivity of transgenic Arabidopsis plants and WT plants to ABA (**A**) growth and germination rate by *CtFLS1* transgenic Arabidopsis plants and WT plants on 1/2 MS medium and 1/2 MS medium containing 0.5 μM, 1 μM and 5 μM ABA. (**B**) Root lengths of CtFLS1 transgenic Arabidopsis plants and WT plants on 1/2 MS medium and 1/2 MS medium containing 0.5 μM, 1 μM, 5 μM, 10 μM, 20 μM ABA. (**C**) Root length statistical analysis. * *p* < 0.05, ** *p* < 0.01.

The role of abscisic acid (ABA) in drought stress response was further investigated by subjecting wild-type, fls mutant, and CtFLS1 transgenic Arabidopsis seeds to varying concentrations (0, 0.5, 1, and 5 μM) of ABA treatments for 7 days, respectively. The results showed that under normal conditions (0 μM ABA), there was no significant difference in seed germination rates between CtFLS1-overexpression lines and WT plants, indicating that CtFLS1 overexpression did not affect germination under non-stress conditions. However, under 0.5 μM ABA treatment, the germination rate of CtFLS1-overexpression lines was substantially higher (50%) compared to WT and fls mutant lines (15%) on the seventh day, suggesting that CtFLS1 overexpression confers enhanced resistance to ABA-induced inhibition of germination (Figure 5A). On the contrary, no significant effect of ABA treatment under 1 and 5 μM was observed. This indicates that CtFLS1-overexpression lines are better equipped to withstand ABA-mediated stress during germination, potentially due to alterations in the ABA signaling pathways. Furthermore, the study assessed the sensitivity of CtFLS1 transgenic lines to ABA during early seedling development. When exposed to 0.5 and 1 μM ABA concentrations, CtFLS1-overexpression lines exhibited improved root growth relative to wild-type and fls mutant lines. This observation suggests that CtFLS1-overexpression lines are less affected by ABA-mediated inhibition of root growth, indicating a potential role of CtFLS1 in modulating ABA responses during seedling establishment (Figure 5B,C). These findings provide valuable insights into the molecular mechanisms underlying the interaction between CtFLS1 and ABA signaling pathways, highlighting CtFLS1 as a potential regulator of ABA-mediated responses to drought stress in plants.

### 2.5. Subcellular Localization of CtFLS1 and CtANS1

**Figure 6:**
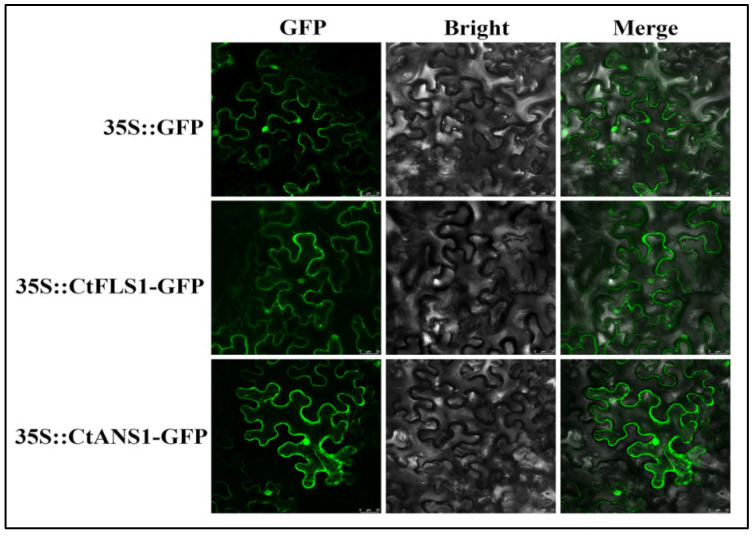
Subcellular localization of CtFLS1 and CtANS1 protein in N. benthamiana leaves. The samples were photographed with scanning electron microscope; bars = 25 μm.

The subcellular localization of CtFLS1 and CtANS1 was investigated by transforming the recombinant plasmids containing GFP-tagged CtFLS1 and CtANS1 into Nicotiana benthamiana leaves, along with a positive control expressing GFP alone (Figure 6). Confocal microscopy analysis revealed that both GFP-tagged CtFLS1 and CtANS1 exhibited similar subcellular localization patterns, appearing predominantly in the cell membrane and nucleus. These observations confirm that CtFLS1 and CtANS1 are localized to cell membrane and nucleus within plant cells, suggesting their potential involvement in essential molecular functions and activities. This subcellular localization data provide valuable insights into the cellular roles of CtFLS1 and CtANS1, laying the foundation for further investigation into their functional significance in safflower physiology and metabolism.

### 2.6. CtFLS1 Interacts with CtANS1

**Figure 7:**
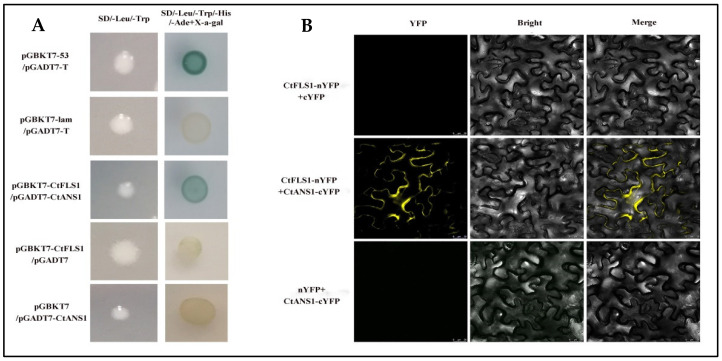
CtFLS1 interaction with CtANS1. (**A**) Yeast two-hybrid analysis. CDS of *CtFLS1* and *CtANS1* were fused with the GAL4-binding domain (pGBKT7-CtFLS1) and the GAL4 activation domain (pGADT7-CtANS1), respectively. Yeast co-transformed with pGBKT7-53 + pGADT7-T served as a positive control, and yeast co-transformed with vectors pGBKT7-Lam + pGADT7-T, pGBKT7-CtFLS1 + pGADT7, and pGBKT7 + pGADT7-CtANS1 served as negative controls. Co-transformed yeast cells were grown on SD/-Leu/-Trp and SD/-Leu/-Trp/-His/-Ade supplementing X-a-gal medium. (**B**) Bimolecular fluorescence complementation (BiFC) assay in *N. benthamiana* leaves. CDS of *CtFLS1* and *CtANS1* were fused to the N-terminal part of YFP and the C-terminal part of YFP, respectively. Bars = 25 μm.

To investigate the potential interaction between CtFLS1 and CtANS1, directed yeast two-hybrid (Y2H) assays were conducted. The coding sequence (CDS) of CtFLS1 was inserted into the pGBKT7 vector (pGBKT7-CtFLS1), while the CDS of CtANS1 was fused with the pGADT7 vector (pGADT7-CtANS1). Co-transformation of yeast cells with pGBKT7-CtFLS1 and pGADT7-CtANS1 revealed interaction between CtFLS1 and CtANS1, as evidenced by positive growth on selective media. This interaction was absent in negative control experiments (Figure 7A). Further validation of the CtFLS1-CtANS1 interaction was performed using bimolecular fluorescence complementation (BiFC) assays in Nicotiana benthamiana leaves. CtFLS1 was fused to the N-terminal fragment of yellow fluorescent protein (YFP) as CtFLS1-nYFP, and CtANS1 was fused to the C-terminal fragment of YFP as CtANS1-cYFP. Co-expression of CtFLS1-nYFP and CtANS1-cYFP resulted in the appearance of a yellow, fluorescent signal specifically at the cell membrane, indicative of protein–protein interaction. No such signal was observed in control experiments (Figure 7B). These findings provide conclusive evidence that CtFLS1 interacts with CtANS1, particularly at the cell membrane, suggesting potential functional crosstalk between these proteins in safflower.

### 2.7. Ectopic Expression of CtFLS1 Enhanced Arabidopsis Drought Tolerance

**Figure 8:**
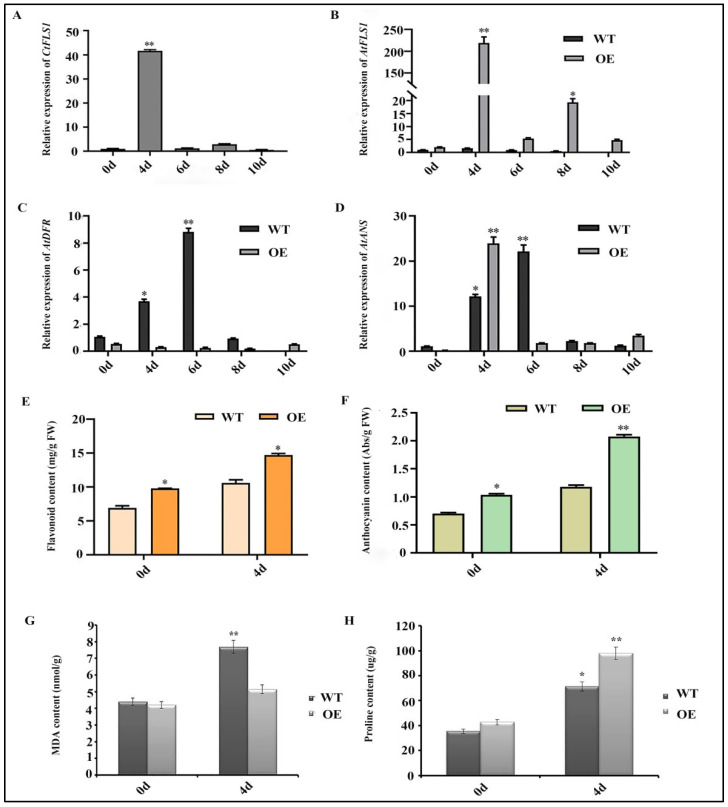
*CtFLS1* transgenic Arabidopsis lines exhibit enhanced drought tolerance. (**A**) The expression patterns of *CtFLS1* in transgenic Arabidopsis after dehydration treatment at five time points from 0 to 10 days, the 0 d dehydration treatment was used as the control (relative expression level = 1). (**B**) Relative expression level of *AtFLS1*, (**C**) *AtDFR,* and *AtANS* (**D**) in WT and *CtFLS1*-overexpressed Arabidopsis under 0, 4, 6, 8, and 10 days of dehydration treatment. (**E**) Total flavonoids content, (**F**) anthocyanin content, (**G**) MDA content, and proline content (**H**) in the wild-type and *CtFLS1*-overexpressed Arabidopsis plant after 0 and 4 days of dehydration treatment. Data represent means of three replicates, and asterisks indicate significant differences, * *p* < 0.05, ** *p* < 0.01.

According to the results of germination and root lengths assay, it is indicated that *CtFLS1* confers drought tolerance to Arabidopsis. To further elucidate its mechanism, the expression patterns of the *CtFLS1* gene in transgenic Arabidopsis were analyzed by qRT-PCR over a time course of dehydration treatment. The results showed that the mRNA abundance of the *CtFLS1* gene reached its peak of approximately 41.35-fold at 4 days compared with day 0, but its expression level rapidly decreased after 6 days (Figure 8A). These results indicated that transcription of *CtFLS1* gene can be influenced by dehydration treatment. Next, the expression patterns of three genes (*AtFLS1*, *AtDFR* and *AtANS*) in the flavonoid biosynthesis pathway in the transgenic and WT Arabidopsis under dehydration treatment and different periods of time were analyzed by qRT-PCR. The results indicated that the expression levels of *AtFLS1* and *AtANS* gene were higher in transgenic Arabidopsis than in the WT plants under dehydration treatment for 4 days, while the *AtDFR* gene was not significantly different in transgenic plants under drought conditions (Figure 8B–D).

We next measured the content of total flavonoid, anthocyanin, MDA and proline in the wild-type and transgenic Arabidopsis plants under dehydration treatment for 0 and 4 days, respectively. The results showed that the total flavonoid and anthocyanin content were significantly higher in transgenic Arabidopsis lines compared to wild-type plants under dehydration treatment for 4 days (Figure 8E,F). Meanwhile, the MDA level was considerably lower in the transgenic plants than in the wild-type plants after drought treatment (WT 7.685 nmol/g, OE 5.143 nmol/g) (Figure 8G), and the content of proline was significantly increased in the transgenic plants compared to the wild-type plants after drought treatment (WT 71.382 μg/g, OE 97.965 μg/g) (Figure 8H). Taken together, our results suggest that *CtFLS1* could effectively enhance the drought tolerance in transgenic Arabidopsis.

## 3. Discussion

The flavonoid biosynthetic pathway, crucial for plant development and stress response, is orchestrated by a network of enzymes, including flavonol synthases (FLS) and anthocyanidin synthases (ANS) [31,32,33]. Nevertheless, the pathway remains highly complex, with each plant species exhibiting a distinct flavonoid profile. Furthermore, F3H, FNSI, FLS, and ANS are all members of the 2-oxoglutarate-dependent dioxygenase (2-ODD) family of proteins, sharing identical conserved domains [34,35,36]. Understanding this complex pathway of flavonoid biosynthesis across diverse plant species is essential for unraveling their functional significance in plant physiology and stress responses [35,37,38]. In our study, we identified and characterized CtFLS1 in safflower, which shed light on its role as a key regulator in flavonoid metabolism and drought stress tolerance. The bioinformatics analysis revealed that CtFLS1 protein possesses 2-ODD superfamily conserved motifs (HxDxnH and RxS motifs) for binding ferrous iron and 2-OG, respectively [39]. In addition, CtFLS1 protein also possesses two FLS-specific motifs, PxxxIRxxxEQP and SxxTxLVP. These two motifs were previously proposed to be important for FLS activity and used to distinguish FLS from other 2-ODD superfamily members [40,41]. This conservation underscores the evolutionary importance and functional relevance of CtFLS1 in safflower flavonoid biosynthesis, corroborating previous findings in different plant species including moss species [30], *Brassica napus* [29], and *Euphorbia kansui* [31].

The diverse subcellular localization of FLS genes among different crops indicates that the machinery responsible for flavonol biosynthesis is active in various cellular compartments [42]. This suggests that flavonols may exert their regulatory influence on gene transcription within these distinct subcellular environments. For example, in cotton, nine GhFLS proteins predominantly localize to the cytoplasm, chloroplast, and nucleus: three are specifically situated in the chloroplast, eight are predominantly cytoplasmic, and three are confined to the nucleus [43]. In rice, *OsFLS1* and *OsFLS3* are both cytoplasmic, whereas *OsFLS1* localizes to the nucleus [44]. Similarly, *AtFLS1* in Arabidopsis is found to be located in both the nucleus and cytoplasm [41]. Interestingly, the subcellular localization results showed that *CtFLS1* and *CtANS1* were found to be located in cell membrane and nucleus within plant cells, suggesting their potential involvement in essential molecular functions and activities. Moreover, our expression analysis of *CtFLS1* during different flowering stages of safflower suggests important insights into its spatiotemporal regulation and its potential correlation with total flavonoid content. The observed upregulation of *CtFLS1* transcript levels coinciding with increased flavonoid accumulation suggests a regulatory role for *CtFLS1* in modulating flavonoid biosynthesis in safflower. This correlation underscores the significance of *CtFLS1* as a potential target for manipulating flavonoid content in safflower cultivars, offering implications for enhancing nutritional value and stress resilience.

Plants exhibit complex mechanisms to adapt to various environmental stimuli, encompassing temperature fluctuations, water availability, nutrient status, and hormonal cues [45,46]. These factors play pivotal roles in shaping the distribution and accumulation patterns of flavonol compounds [8,47]. Plant hormones are widely recognized for their pivotal roles in orchestrating growth, development, and responses to diverse environmental stresses [48,49,50]. They achieve this by finely regulating the expression of genes associated with these processes. Furthermore, these phytohormones intricately regulate the biosynthesis of secondary metabolites, notably flavonols, within plants [51,52,53]. Prior studies have demonstrated that the expression of FtFLS1 is suppressed by salicylic acid (SA) and abscisic acid (ABA), whereas FtFLS2 is unaffected by ABA but induced by SA in buckwheat [54]. Additionally, FLS genes, potentially involved in flavonoid formation, exhibit heightened expression levels after a 2-h treatment with methyl jasmonate (MeJA) [55,56]. Likewise, the FLS gene in leaves exhibits peak expression levels in plants treated with methyl jasmonate (MeJA) and salicylic acid (SA) during the vegetative growth stage [24,57,58]. In this study, our transgenic Arabidopsis experiments elucidate the functional consequences of CtFLS1 overexpression on flavonoid metabolism and stress tolerance. Our results showed that the ectopic expression of CtFLS1 resulted in a significant increase in total flavonoid content, accompanied by altered expression patterns of key genes in the flavonoid biosynthetic pathway. Importantly, the observed downregulation of anthocyanin biosynthesis genes, in conjunction with elevated flavonol levels, highlights the pivotal role of CtFLS1 in redirecting flavonoid flux towards flavonol synthesis. Moreover, our investigation into the stress response mechanisms mediated by CtFLS1 provides intriguing insights into its potential role in abiotic stress tolerance. The enhanced osmotic stress tolerance was observed in CtFLS1-overexpressing Arabidopsis lines. Additionally, our findings on reduced sensitivity to ABA-induced inhibition of germination and root growth suggest a potential regulatory role for CtFLS1 in modulating ABA signaling pathways. These observations underscore the multifaceted roles of CtFLS1 in plant stress adaptation and highlight its potential as a target for engineering stress-tolerant crops. The identification of CtFLS1 as a key player in safflower flavonoid metabolism offers promising avenues for crop improvement and stress resilience breeding strategies, with implications for sustainable agriculture and food security.

## 4. Materials and Methods

### 4.1. Plant Materials

Seeds of safflower (Jihong No.1) were cultivated in the experimental field at the Jilin Agricultural University (Changchun, Jilin province, 43°53′ N,125°19′ E). The flower petals were collected at different flowering developmental stages, including bud (75 days after sowing), initial (82 days after sowing), complete (89 days after sowing), and fading (99 days after sowing). All collected flower petals were immediately frozen in liquid nitrogen and stored at −80 °C for future analysis.

### 4.2. Bioinformatics Analysis of CtFLS1

Full-length sequences of *CtFLS1* open reading frame (ORF) were obtained from NCBI (https://www.ncbi.nlm.nih.gov (accessed on 15 May 2024)) (GenBank accession number: KP300884.1) and analyzed using online bioinformatics tools. The physicochemical properties of CtFLS1, including deduced amino acid sequences, theoretical isoelectric point (pI), and predicted molecular weight (MW), were calculated using the online software ExPasy (https://www.expasy.org/ (accessed on 15 May 2024)). The SWISS-MODEL server prepared the three-dimensional (3D) structure of CtFLS1 protein (https://swissmodel.expasy.org/ (accessed on 15 May 2024)). The homologous sequences of CtFLS1 in different species were obtained from the NCBI databases using Blastp, followed by multiple sequence alignments using the DNAMAN software (DNAMAN Version 9). Finally, the phylogenetic tree was constructed using MEGA-X software (MEGA-X Version 10.1.8) with the neighbor-joining (NJ) method and the bootstrap analysis for 1000 replicate fields.

### 4.3. Expression Analysis of CtFLS1 Gene in Safflower

To analyze the relative expression level of the *CtFLS1* gene, the total RNA was extracted from bud (75 days after sowing), initial (82 days after sowing), full (89 days after sowing), and fading flowering stages (99 days after sowing), respectively, using RNAiso Plus reagent (TaKaRa, Beijing, China) according to the manufacturer’s protocol. The integrity and concentration of total RNA were analyzed using 1% agarose gel electrophoresis and NanoDrop 2000 (ThermoFisher Scientific, Beijing, China). First-strand cDNA was synthesized from 1 µg of total RNA using PrimeScript™ RT Reagent Kit (TaKaRa, Dalian, China). The cDNA was diluted 20-fold and used as the template for qRT-PCR. The qRT-PCR was performed with SYBR^®^ Premix Ex Taq™ (Takara, Dalian, China) using the Stratagene Mx3000P thermocycler (Agilent Technologies Inc, California, United States) system following the manufacturer’s instructions. Specific qRT-PCR primers of the CtFLS1 gene were designed with Premier 5, and the C. tinctorius 18 s *ribosomal RNA* gene was used as the internal reference gene in qRT-PCR analysis. The 2^−ΔΔCt^ method was used to calculate the relative expression level of the *CtFLS1* gene in four flowering developmental stages. Specific qRT-PCR primers used in the experiment are listed in Supplementary Table S1. Three biological and three technical replicates were performed in gene expression analysis.

### 4.4. Generation of Transgenic Arabidopsis

To obtain *CtFLS1* transgenic *Arabidopsis* plants, the complete coding sequence of the *CtFLS1* gene was cloned into the plant expression vector pBasta containing the selectable marker gene (BAR), with *Spe I* and *Xba I* restriction sites under the control of CaMV35S promoter. Then, the recombinant plasmid pBasta-*CtFLS1* was transformed into EHA105 competent cell, the *CtFLS1* overexpressing transgenic Arabidopsis lines were generated using the Agrobacterium-mediated floral dip method. T1 transgenic Arabidopsis plants were screened by spraying with 1% Basta solution, and the same selection method was used to obtain T2 generation and T2 transgenic lines accord with 3:1 rate, two T3 homozygous lines (OE1 and OE7) with the highest expression levels of *CtFLS1* were selected by qRT-PCR for further functional analysis.

### 4.5. Expression Analysis in Transgenic Arabidopsis

To analyze the endogenous genes expression of flavonoids’ biosynthesis pathway, including four structural genes (*AtCHS*, *AtFLS1*, *AtDFR,* and *AtANS*) and four transcription factors (TFs) (*AtMYB11*, *AtMYB12*, *AtMYB111,* and *AtMYB113*), the total RNAs were extracted from the wild type and two *CtFLS1*-OE lines (OE1 and OE7) using the previously described protocol. The first-strand cDNAs were synthesized using the PrimeScript™ RT Reagent Kit, and the qRT-PCR was carried out using SYBR^®^ Premix Ex Taq™ on a Mx3000P (Agilent) real-time PCR detection system. The specific primers used in this experiment are listed in Supplementary Table S1. The *18s ribosomal RNA* gene (AT5G38720.1) was used as an internal reference gene. The relative gene expression level was analyzed using the 2^−ΔΔCt^ method. Three biological and three technical replicates were performed for qRT-PCR.

### 4.6. DPBA Staining

Flavonol accumulation in leaves was visualized using diphenylboric acid 2-aminoethyl ester (DPBA) staining, following a modified protocol described by [59]. Firstly, leaves from wild-type (WT) Arabidopsis and two transgenic lines underwent treatment with absolute alcohol until they became colorless, effectively removing chlorophyll and other interfering pigments. Subsequently, the colorless leaves were submerged in MES-KCL buffer supplemented with 0.25% (*w*/*v*) DPBA and 0.01% (*v*/*v*) Triton X-100 for 1 h under dark conditions at 25 °C to facilitate the specific binding of DPBA to flavonols present in the leaves. Following staining, the leaves were washed three times with MES-KCL buffer to remove excess staining solution and Triton X-100. The stained leaves were then transferred onto glass slides, mounted in water, and visualized using an inverted microscope equipped with appropriate fluorescence filters. The resulting fluorescence signal emitted by DPBA-bound flavonols was observed and documented, allowing for the assessment of flavonol accumulation patterns in the leaves. This staining procedure provides valuable insights into the spatial distribution and abundance of flavonols in response to genetic manipulation or environmental stimuli, aiding in the understanding of flavonoid metabolism and its regulation in plants.

### 4.7. Measurement of Total Flavonoids and Anthocyanin Levels

The extraction and measurement of total flavonoids were performed as previously described by [24]. Briefly, flavonoids in the sample were extracted with 65% (*v*/*v*) methanol for 1.5 h at 80 °C. The extracts were centrifuged at 4000 rpm for 10 min, and 100 µL supernatants were transferred to new plastic tubes containing 10 µL NaNO_2_ (5%, *w*/*v*) for 6 min (mins) at room temperature. Subsequently, 10 µL Al(NO_3_)_3_ (10%, *w*/*v*) was added to each tube and incubated for 6 min. Next, the reaction was terminated by adding 30 µL NaOH (4%, *w*/*v*). The absorbance at 510 nm was recorded using a Microplate Reader (TECAN, Mannedorf, Switzerland). Different concentrations of rutin were used as a standard for making standard curves.

### 4.8. Germination and Root Length Assays of Transgenic Arabidopsis

For germination assay, the seeds of wild type, *fls* mutant, and *CtFLS1* overexpression transgenic lines of Arabidopsis were sterilized and sown on 1/2 MS medium containing 0, 100, 200, 300, and 400 mM mannitol or 0, 0.5, 1, and 5 µM ABA, respectively [60]. All the plates were placed in the dark at 4 °C for 3 days (d) and then transferred to a growth room for 7 d. The seed germination rate was calculated daily, and photos were taken on the 7th day. At least 55 seeds were sown for each replicate of three parallel replicates. For root elongation assay, five-day-old seedlings were transferred to 1/2 MS medium containing 0, 100, 200, 300, and 400 mM mannitol or 0, 0.5, 1, 5, 10, and 20 µM ABA for 10 d. The primary root lengths were measured, and pictures were taken on 10th day. All the experiments were repeated three times.

### 4.9. Expression Analysis of CtFLS1 and Relevant Genes under Drought Stress

Two-week-old wild-type (WT) and transgenic Arabidopsis plants were subjected to dehydration treatment, and leaf samples were collected at various time points (0 d, 4 d, 6 d, 8 d, and 10 d). The collected samples were immediately frozen in liquid nitrogen and stored at −80 °C until further analysis. Total RNA was extracted, and the first strand cDNA templates were prepared using reverse transcriptase enzyme. Then, specific primers were designed using Premier 3 software, and qRT-PCR was employed to assess the expression levels of CtFLS1, as well as key genes involved in the flavonoid pathway. The Arabidopsis 18S ribosomal RNA gene was used as an internal reference gene for normalization purposes, ensuring accurate quantification of gene expression levels.

### 4.10. Measurements of MDA and Proline Contents

Fresh leaf samples were collected from both transgenic and wild-type (WT) plants under both normal and drought treatment conditions to assess changes in physiological parameters. The quantification of malondialdehyde (MDA) and proline content was performed using commercially available detection kits (Solarbio, Beijing, China), following the manufacturer’s instructions. For MDA content determination, leaf samples were homogenized in ice-cold MDA assay buffer, and the resulting supernatant was mixed with thiobarbituric acid (TBA) reagent to form a colored MDA-TBA adduct. After incubation and centrifugation, the absorbance of the supernatant was measured at the appropriate wavelength using a spectrophotometer. Similarly, for proline content quantification, leaf samples were homogenized in proline assay buffer, and the resulting supernatant was mixed with the proline assay reagent to form a colored complex. The absorbance of the reaction mixture was measured at the appropriate wavelength. Standard curves prepared using known concentrations of MDA and proline standards were used to calculate the respective contents in the leaf samples. Statistical analysis was performed to determine significant differences in MDA and proline content between transgenic and WT plants under normal and drought treatment conditions.

### 4.11. Subcellular Localization

The cDNA of CtFLS1 and CtANS1 without stop codons were subcloned into the pCAMBIA-1302 vector to generate the CtFLS1-GFP and CtANS1-GFP fusion protein, respectively. The empty vector containing only the GFP sequence was used as a control. These constructs were transformed into EHA105 competent cell using the freeze-thaw method and infiltrated into leaf epidermal cells of *N. benthamiana* [61]. After 48–96 h, the GFP fluorescence signals were observed using confocal laser scanning microscopy (Leica, Solms, Germany).

### 4.12. Yeast Two-Hybrid (Y2H) Assays

To verify the interaction between CtFLS1 and CtANS1 proteins, the complete cDNA of CtFLS1 was cloned into the pGBKT7 vector, while the complete cDNA of CtANS1 was inserted into the pGADT7 vector. The recombinant plasmids were co-transformed into yeast-competent cells (Y2H Gold strains) using the PEG/LiAC method. The transformants were cultured on the selective medium lacking Leu and Trp (SD/-Leu/-Trp) at 30 °C for 3 d. Then, successfully transformed yeast cells were screened on the medium lacking Ade, His, Leu, and Trp (SD/-Ade/-His/-Leu/-Trp) with X-alpha-gal to identify the interaction of CtFLS1with CtANS1.

### 4.13. Bimolecular Fluorescence Complementation Assays

For the Biomolecular Fluorescence Complementation (BiFC) assay, the cDNA of CtFLS1 and CtANS1 were recombined into 35S-pxy106-YFPN and 35S-pxy104-YFPC, respectively. These fusion vectors were separately transformed into *A. tumefaciens* strain EHA105 and co-injected into N. benthamiana leaves. After incubating in darkness for 2–4 d, the YFP fluorescence signal was observed using confocal laser scanning microscopy (Leica, Solms, Germany).

## 5. Conclusions

In this study, we have demonstrated the pivotal role of the CtFLS1 gene as a positive regulator of drought tolerance in transgenic Arabidopsis plants. Our findings reveal that CtFLS1 modulates the flavonoid biosynthesis pathway, leading to increased levels of total flavonoids and anthocyanins, which contribute to enhanced drought tolerance. Furthermore, our investigation uncovered a novel interaction between CtFLS1 and CtANS1 proteins in safflower, shedding light on potential mechanisms underlying drought response in plants. These results underscore the importance of CtFLS1 in mediating drought resilience and highlight the significance of the flavonoid pathway in plant stress adaptation. Moving forward, our study provides important insights into further exploration of the molecular mechanisms underlying CtFLS1-mediated drought tolerance and the broader implications for crop improvement strategies. Further elucidation of these pathways in the future holds promise for enhancing agricultural sustainability and resilience in the face of climate change challenges.

## Data Availability

All data generated or analyzed during this study are included in the published article and supplementary files.

## Supplementary Materials

The following supporting information can be downloaded at: https://www.mdpi.com/article/10.3390/ijms25105546/s1.
