# Supplementary material for: Safflower CtFLS1-Induced Drought Tolerance by Stimulating the Accumulation of Flavonols and Anthocyanins in Arabidopsis thaliana
Source: Int J Mol Sci. 2024 May 19;25(10):5546. doi: 10.3390/ijms25105546 (PMC11122397; doi:10.3390/ijms25105546)
Supplement: Supplementary file 1 [file ijms-25-05546-s001.zip › ijms-2982822-supplementary.pdf]

**Table S1.** Primers used in qRT-PCR analysis in this study.

| Primer name | Sequence (5’-3’) |
|---|---|
| qRT-*CtFLS1*-F | TGAAACAAGTGGTCCACCCAC |
| qRT-*CtFLS1*-R | ACCATAGCCAAGCCTGCAAA |
| qRT-*AtCHS*-F | AGAAGTTCAAGCGCATGTGC |
| qRT-*AtCHS*-R | AGAGAAGGAGCCATGTAAGCAC |
| qRT-*AtFLS1*-F | TGCAGTGCATGTGAAGAAGC |
| qRT-*AtFLS1*-R | CGAGACCTTCTTTCAACGCATC |
| qRT-*AtDFR*-F | ATTTGCCAAACGCCAAGACG |
| qRT-*AtDFR*-R | TGTTGCCACGTGGAAAACAC |
| qRT-*AtANS*-F | TGCAAACGATCAAGCCACTG |
| qRT-*AtANS*-R | TTGTCCACTCGCGTTGTTAG |
| qRT-*AtMYB11*-F | TTGTCGAGGCTGTTAGATGGAG |
| qRT-*AtMYB11*-R | ACAAAAGCCAAGCAGCCATC |
| qRT-*AtMYB12*-F | ATGACGAACGCTTCTTCAGC |
| qRT-*AtMYB12*-R | TGGTGCAGACGTTTTCTTCG |
| qRT-*AtMYB111*-F | GTCAAGTTGCATTCCCTTCTCG |
| qRT-*AtMYB111*-R | TTTTGCGGCTGAGATGTGAG |
| qRT-*AtMYB113*-F | TTGATCGCTGGTAGATTGCC |
| qRT-*AtMYB113*-R | AGCATCGTTCATCGTGCTTC |
| 18s *rRNA*-F | GAGAAACGGCTACCACATCCAA |
| 18s *rRNA*-R | TCGTTTGAGCCCGGTATTGTTA |

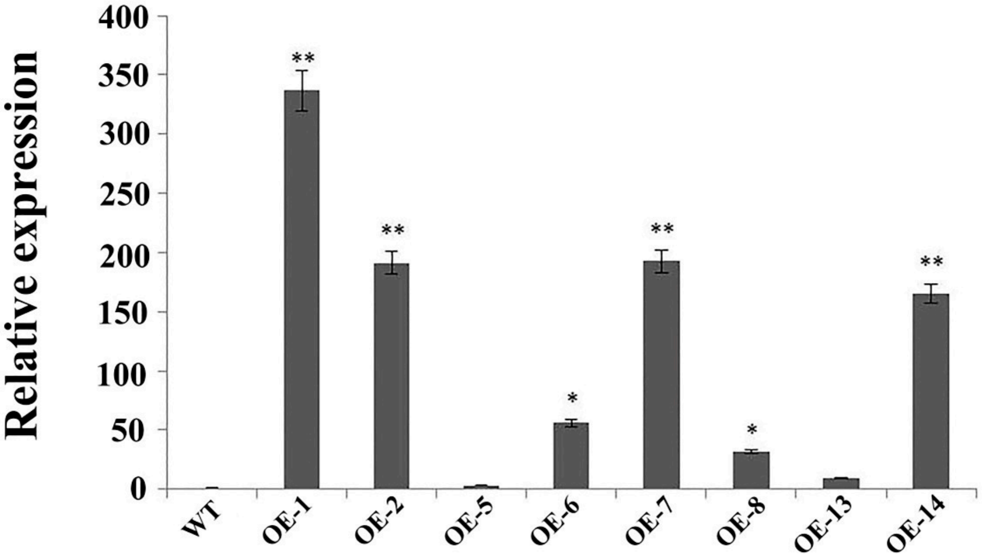


**Figure S1.** The relative expression of *CtFLS1* gene in different *CtFLS1* transgenic *Arabidopsis* lines. Significance analysis t test： *P<0.05, **P<0.01.
